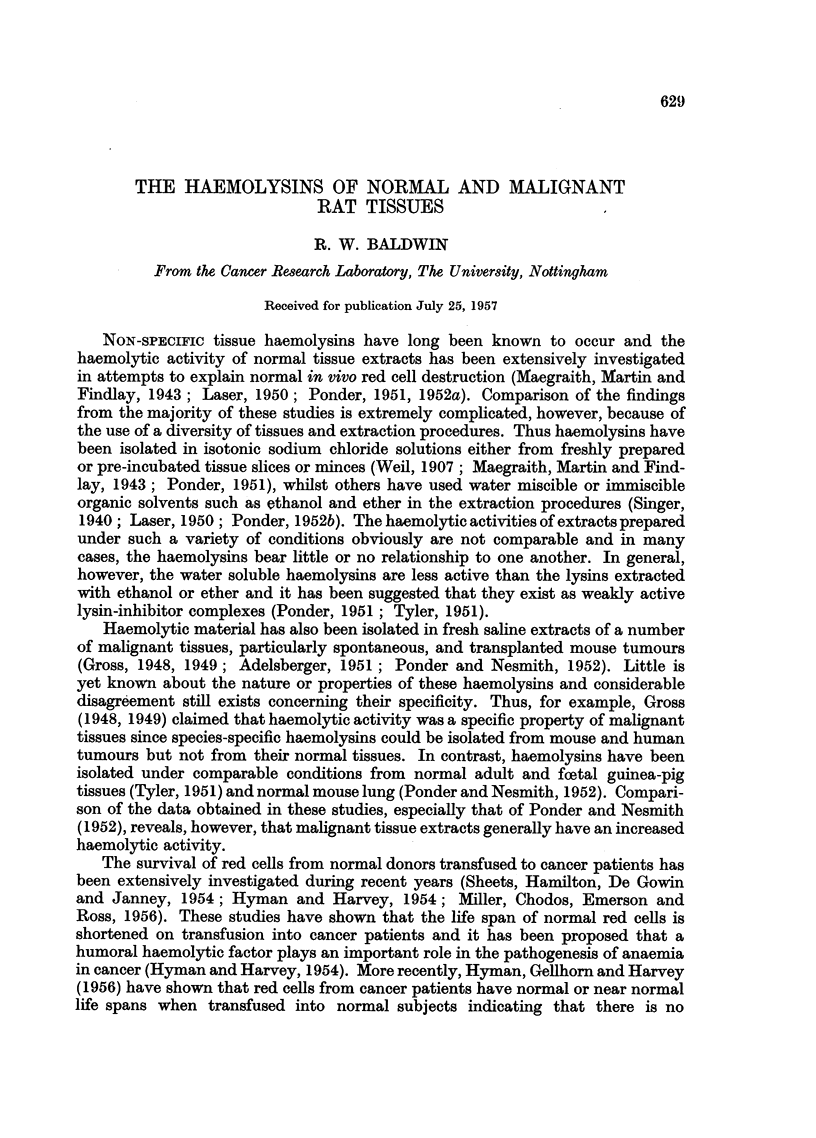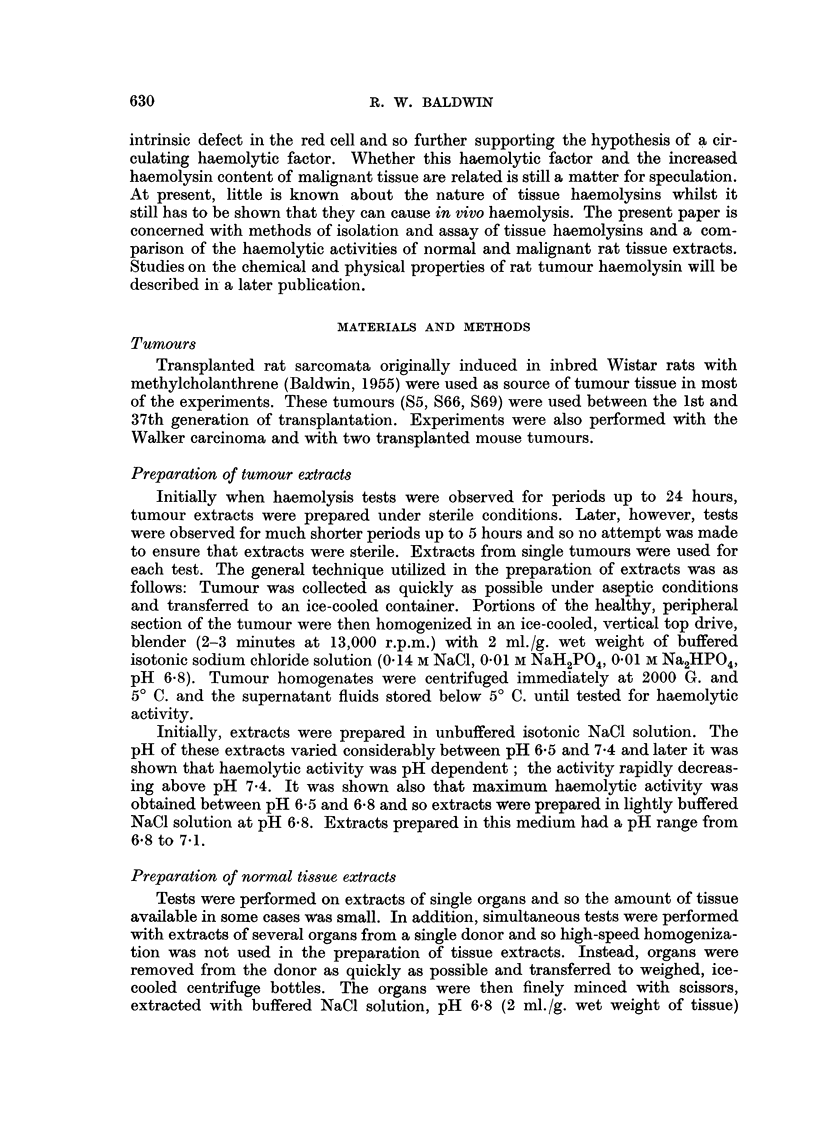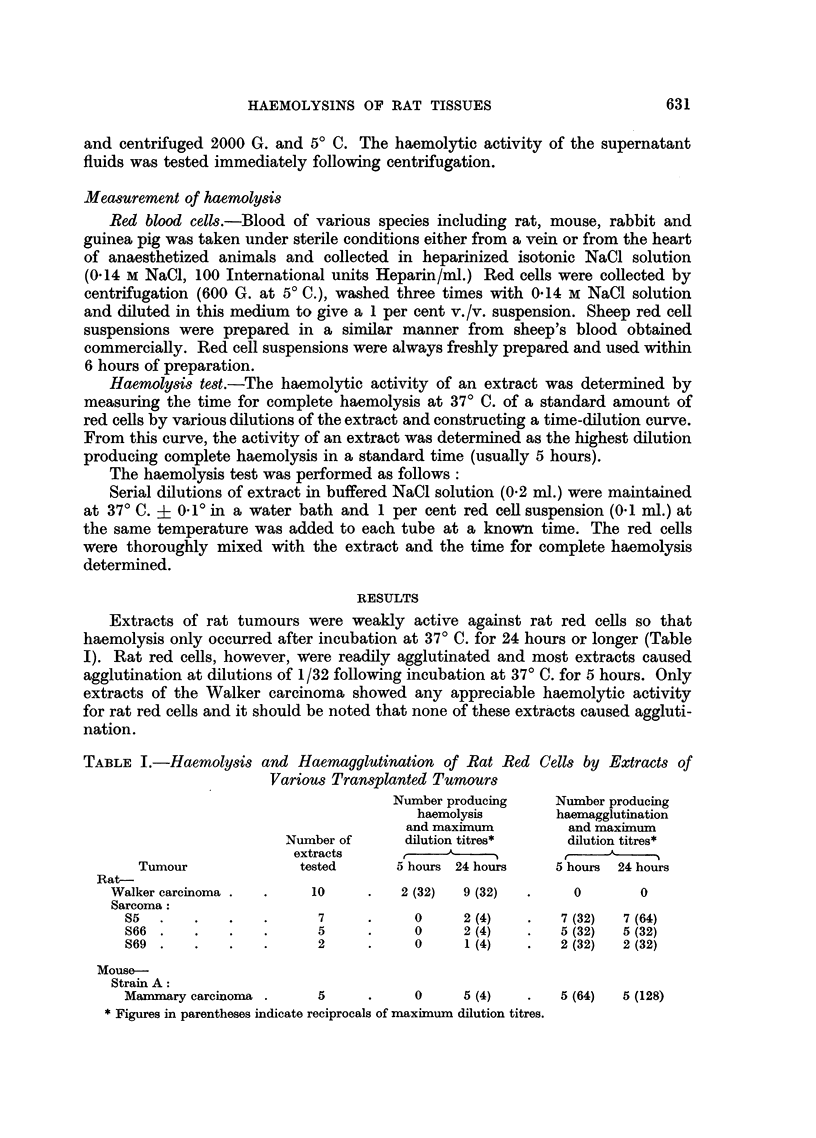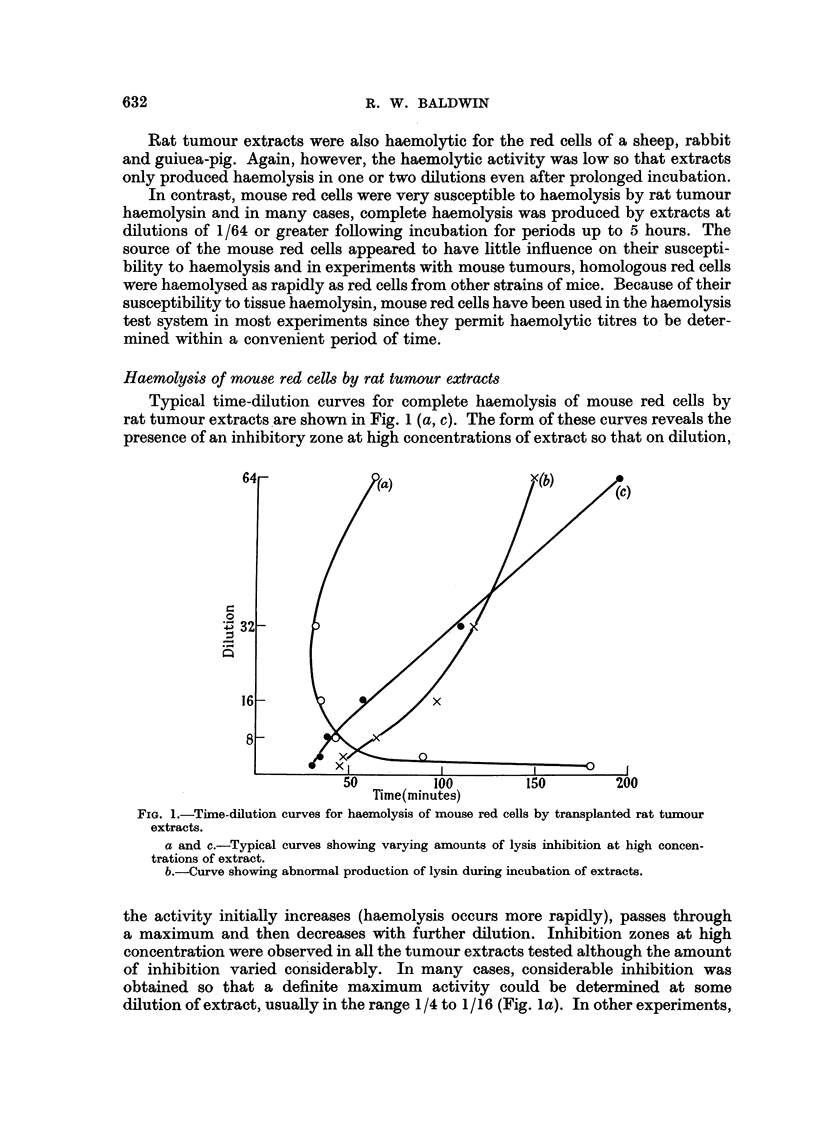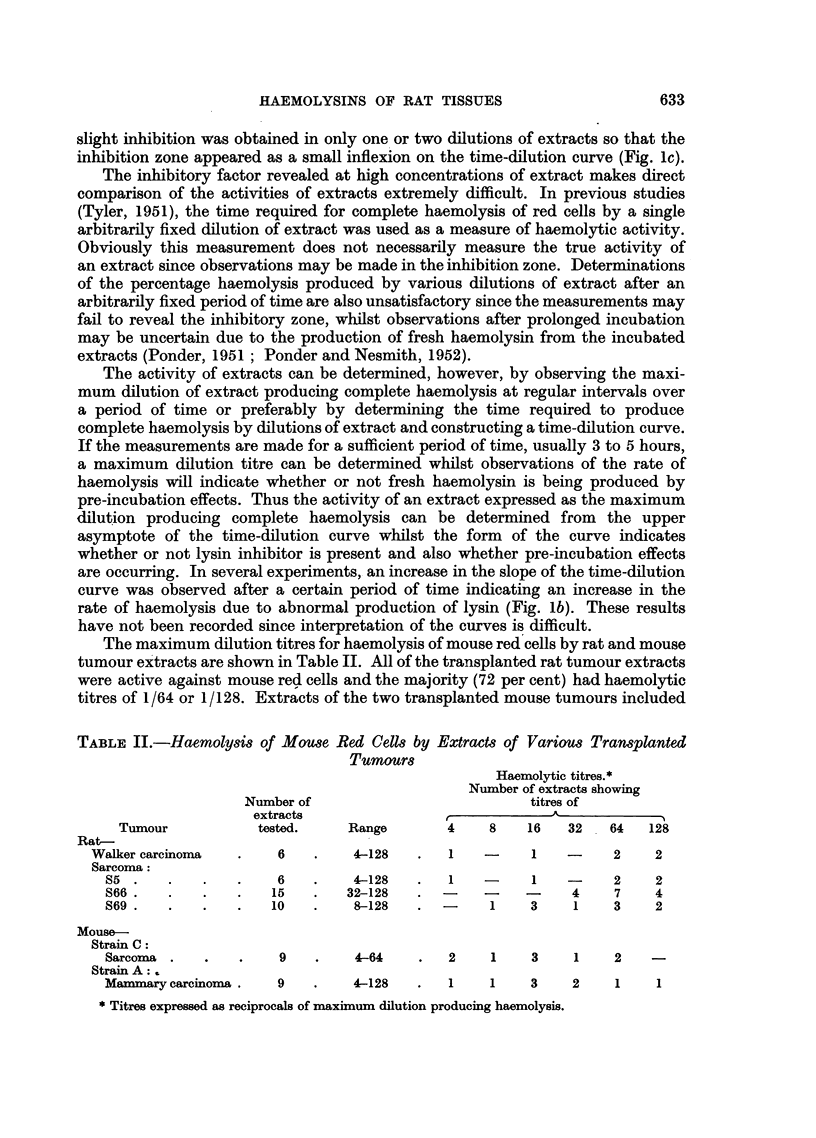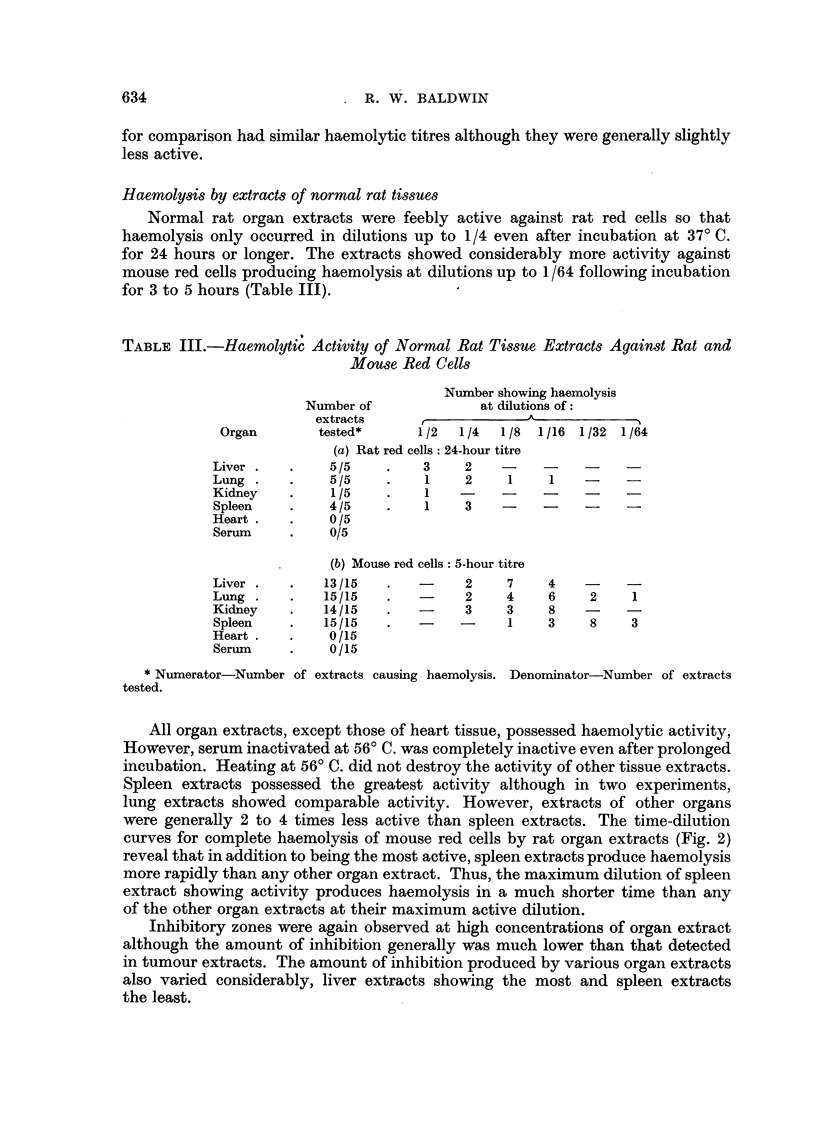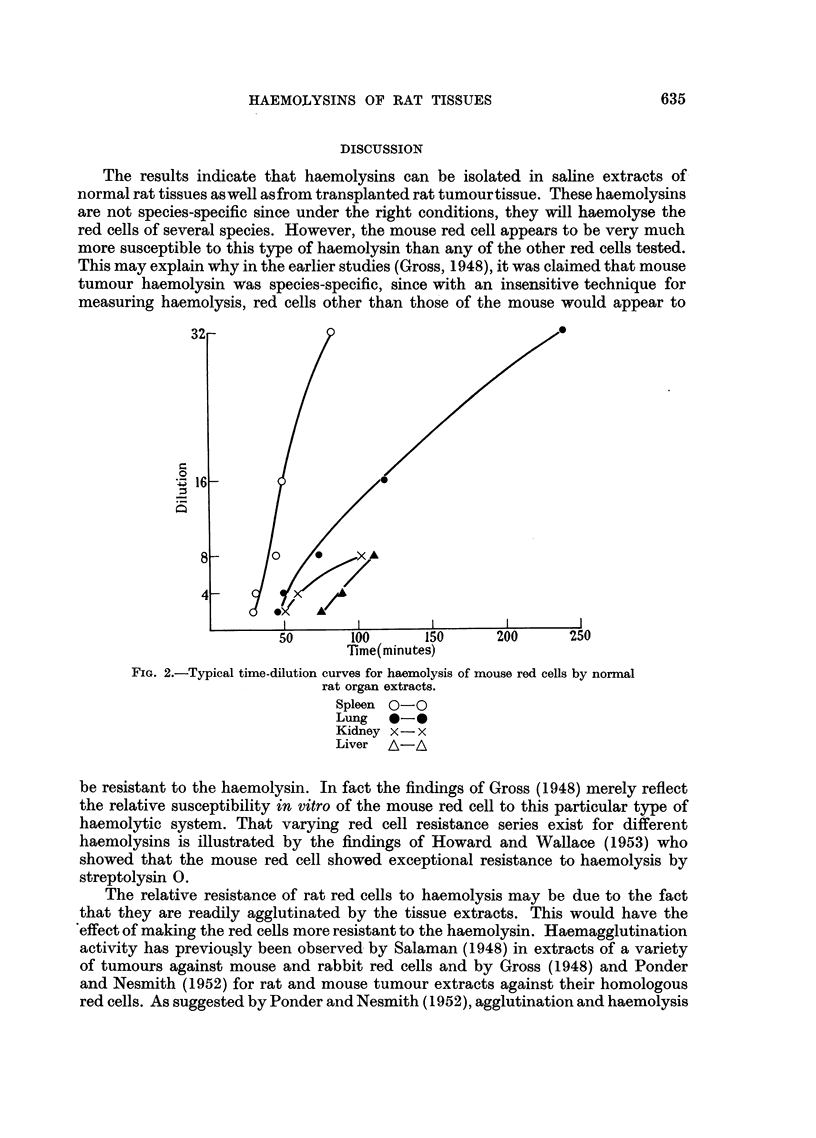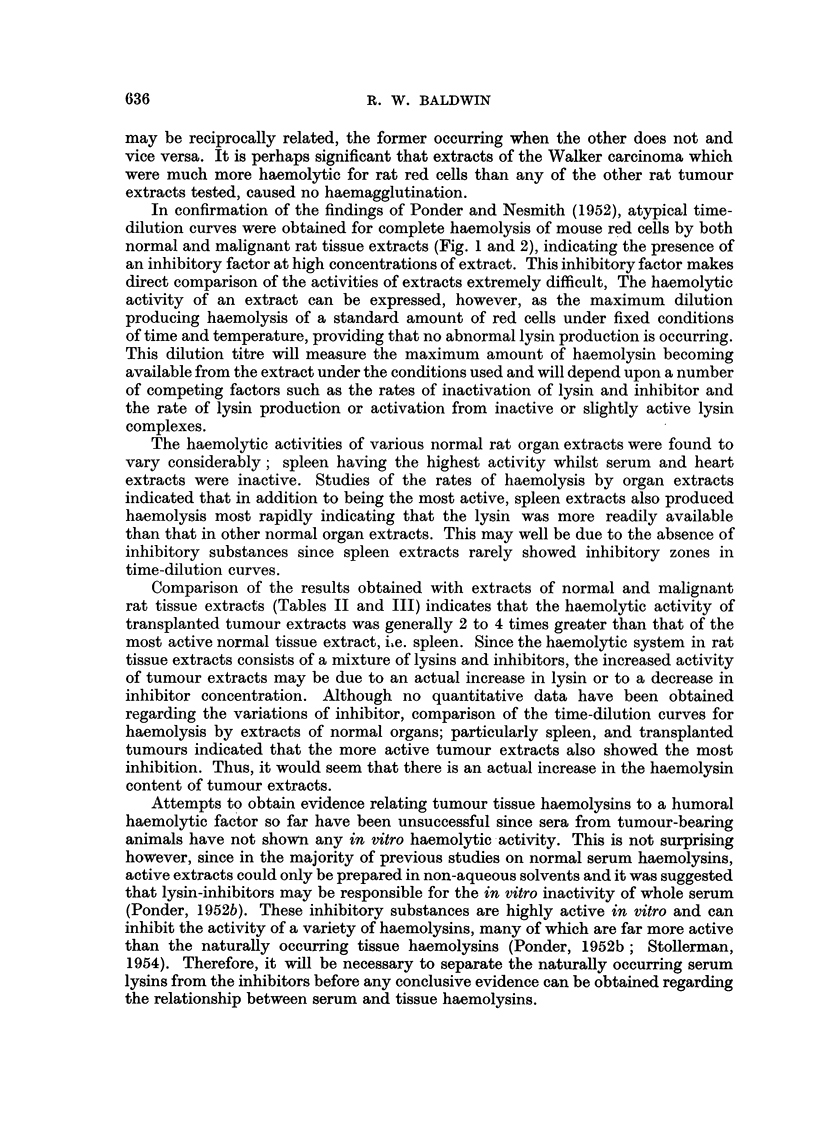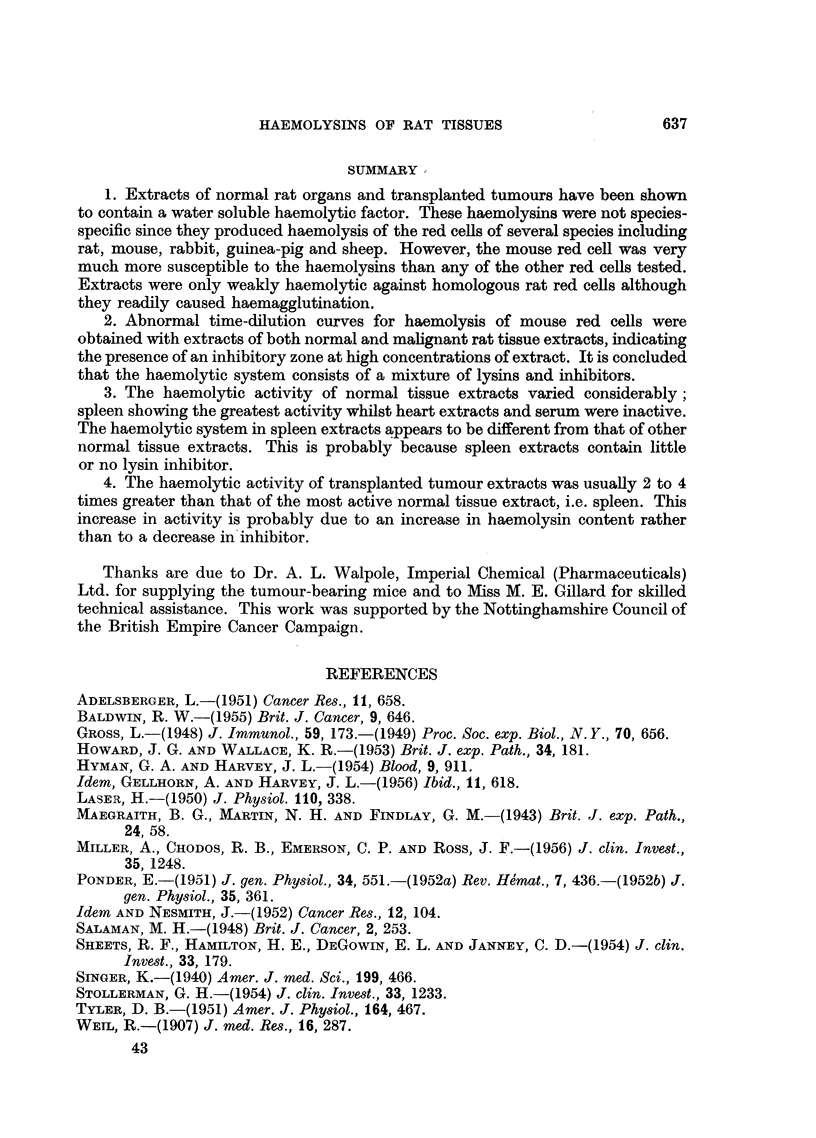# The Haemolysins of Normal and Malignant Rat Tissues

**DOI:** 10.1038/bjc.1957.77

**Published:** 1957-12

**Authors:** R. W. Baldwin


### THE HAEMOLYSINS OF NORMAL AND MALIGNANT RAT TISSUES

R. W. BALDWIN

*From the Cancer Research Laboratory, The University, Nottingham*

Received for publication July 25, 1957

NON-SPECIFIC tissue haemolysins have long been known to occur and the haemolytic activity of normal tissue extracts has been extensively investigated in attempts to explain normal *in vivo* red cell destruction (Maegraith, Martin and Findlay, 1943 ; Laser, 1950 ; Ponder, 1951, 1952*a*). Comparison of the findings from the majority of these studies is extremely complicated, however, because of the use of a diversity of tissues and extraction procedures. Thus haemolysins have been isolated in isotonic sodium chloride solutions either from freshly prepared or pre-incubated tissue slices or minces (Weil, 1907 ; Maegraith, Martin and Findlay, 1943 ; Ponder, 1951), whilst others have used water miscible or immiscible organic solvents such as ethanol and ether in the extraction procedures (Singer, 1940 ; Laser, 1950 ; Ponder, 1952*b*). The haemolytic activities of extracts prepared under such a variety of conditions obviously are not comparable and in many cases, the haemolysins bear little or no relationship to one another. In general, however, the water soluble haemolysins are less active than the lysins extracted with ethanol or ether and it has been suggested that they exist as weakly active lysin-inhibitor complexes (Ponder, 1951 ; Tyler, 1951).

Haemolytic material has also been isolated in fresh saline extracts of a number of malignant tissues, particularly spontaneous, and transplanted mouse tumours (Gross, 1948, 1949 ; Adelsberger, 1951 ; Ponder and Nesmith, 1952). Little is yet known about the nature or properties of these haemolysins and considerable disagreement still exists concerning their specificity. Thus, for example, Gross (1948, 1949) claimed that haemolytic activity was a specific property of malignant tissues since species-specific haemolysins could be isolated from mouse and human tumours but not from their normal tissues. In contrast, haemolysins have been isolated under comparable conditions from normal adult and fœtal guinea-pig tissues (Tyler, 1951) and normal mouse lung (Ponder and Nesmith, 1952). Comparison of the data obtained in these studies, especially that of Ponder and Nesmith (1952), reveals, however, that malignant tissue extracts generally have an increased haemolytic activity.

The survival of red cells from normal donors transfused to cancer patients has been extensively investigated during recent years (Sheets, Hamilton, De Gowin and Janney, 1954 ; Hyman and Harvey, 1954 ; Miller, Chodos, Emerson and Ross, 1956). These studies have shown that the life span of normal red cells is shortened on transfusion into cancer patients and it has been proposed that a humoral haemolytic factor plays an important role in the pathogenesis of anaemia in cancer (Hyman and Harvey, 1954). More recently, Hyman, Gellhorn and Harvey (1956) have shown that red cells from cancer patients have normal or near normal life spans when transfused into normal subjects indicating that there is no intrinsic defect in the red cell and so further supporting the hypothesis of a circulating haemolytic factor. Whether this haemolytic factor and the increased haemolysin content of malignant tissue are related is still a matter for speculation. At present, little is known about the nature of tissue haemolysins whilst it still has to be shown that they can cause *in vivo* haemolysis. The present paper is concerned with methods of isolation and assay of tissue haemolysins and a comparison of the haemolytic activities of normal and malignant rat tissue extracts. Studies on the chemical and physical properties of rat tumour haemolysin will be described in a later publication.

### MATERIALS AND METHODS

### *Tumours*

Transplanted rat sarcomata originally induced in inbred Wistar rats with methylcholanthrene (Baldwin, 1955) were used as source of tumour tissue in most of the experiments. These tumours (S5, S66, S69) were used between the 1st and 37th generation of transplantation. Experiments were also performed with the Walker carcinoma and with two transplanted mouse tumours.

### *Preparation of tumour extracts*

Initially when haemolysis tests were observed for periods up to 24 hours, tumour extracts were prepared under sterile conditions. Later, however, tests were observed for much shorter periods up to 5 hours and so no attempt was made to ensure that extracts were sterile. Extracts from single tumours were used for each test. The general technique utilized in the preparation of extracts was as follows: Tumour was collected as quickly as possible under aseptic conditions and transferred to an ice-cooled container. Portions of the healthy, peripheral section of the tumour were then homogenized in an ice-cooled, vertical top drive, blender (2–3 minutes at 13,000 r.p.m.) with 2 ml./g. wet weight of buffered isotonic sodium chloride solution (0·14 M NaCl, 0·01 M $NaH_2PO_4$, 0·01 M $Na_2HPO_4$, pH 6·8). Tumour homogenates were centrifuged immediately at 2000 G. and 5° C. and the supernatant fluids stored below 5° C. until tested for haemolytic activity.

Initially, extracts were prepared in unbuffered isotonic NaCl solution. The pH of these extracts varied considerably between pH 6·5 and 7·4 and later it was shown that haemolytic activity was pH dependent ; the activity rapidly decreasing above pH 7·4. It was shown also that maximum haemolytic activity was obtained between pH 6·5 and 6·8 and so extracts were prepared in lightly buffered NaCl solution at pH 6·8. Extracts prepared in this medium had a pH range from 6·8 to 7·1.

### *Preparation of normal tissue extracts*

Tests were performed on extracts of single organs and so the amount of tissue available in some cases was small. In addition, simultaneous tests were performed with extracts of several organs from a single donor and so high-speed homogenization was not used in the preparation of tissue extracts. Instead, organs were removed from the donor as quickly as possible and transferred to weighed, ice-cooled centrifuge bottles. The organs were then finely minced with scissors, extracted with buffered NaCl solution, pH 6·8 (2 ml./g. wet weight of tissue)

and centrifuged 2000 G. and 5° C. The haemolytic activity of the supernatant fluids was tested immediately following centrifugation.

### *Measurement of haemolysis*

*Red blood cells.*—Blood of various species including rat, mouse, rabbit and guinea pig was taken under sterile conditions either from a vein or from the heart of anaesthetized animals and collected in heparinized isotonic NaCl solution (0·14 M NaCl, 100 International units Heparin/ml.) Red cells were collected by centrifugation (600 G. at 5° C.), washed three times with 0·14 M NaCl solution and diluted in this medium to give a 1 per cent v./v. suspension. Sheep red cell suspensions were prepared in a similar manner from sheep's blood obtained commercially. Red cell suspensions were always freshly prepared and used within 6 hours of preparation.

*Haemolysis test.*—The haemolytic activity of an extract was determined by measuring the time for complete haemolysis at 37° C. of a standard amount of red cells by various dilutions of the extract and constructing a time-dilution curve. From this curve, the activity of an extract was determined as the highest dilution producing complete haemolysis in a standard time (usually 5 hours).

The haemolysis test was performed as follows :

Serial dilutions of extract in buffered NaCl solution (0·2 ml.) were maintained at 37° C. ± 0·1° in a water bath and 1 per cent red cell suspension (0·1 ml.) at the same temperature was added to each tube at a known time. The red cells were thoroughly mixed with the extract and the time for complete haemolysis determined.

### RESULTS

Extracts of rat tumours were weakly active against rat red cells so that haemolysis only occurred after incubation at 37° C. for 24 hours or longer (Table I). Rat red cells, however, were readily agglutinated and most extracts caused agglutination at dilutions of 1/32 following incubation at 37° C. for 5 hours. Only extracts of the Walker carcinoma showed any appreciable haemolytic activity for rat red cells and it should be noted that none of these extracts caused agglutination.

TABLE I.—*Haemolysis and Haemagglutination of Rat Red Cells by Extracts of Various Transplanted Tumours*

| Tumour | Number of extracts tested | Number producing haemolysis and maximum dilution titres* | | Number producing haemagglutination and maximum dilution titres* | |
|---|---|---|---|---|---|
| | | 5 hours | 24 hours | 5 hours | 24 hours |
| Rat— | | | | | |
| Walker carcinoma | 10 | 2 (32) | 9 (32) | 0 | 0 |
| Sarcoma : | | | | | |
| S5 | 7 | 0 | 2 (4) | 7 (32) | 7 (64) |
| S66 | 5 | 0 | 2 (4) | 5 (32) | 5 (32) |
| S69 | 2 | 0 | 1 (4) | 2 (32) | 2 (32) |
| Mouse— | | | | | |
| Strain A : | | | | | |
| Mammary carcinoma | 5 | 0 | 5 (4) | 5 (64) | 5 (128) |

* Figures in parentheses indicate reciprocals of maximum dilution titres.

Rat tumour extracts were also haemolytic for the red cells of a sheep, rabbit and guiuea-pig. Again, however, the haemolytic activity was low so that extracts only produced haemolysis in one or two dilutions even after prolonged incubation.

In contrast, mouse red cells were very susceptible to haemolysis by rat tumour haemolysin and in many cases, complete haemolysis was produced by extracts at dilutions of 1/64 or greater following incubation for periods up to 5 hours. The source of the mouse red cells appeared to have little influence on their susceptibility to haemolysis and in experiments with mouse tumours, homologous red cells were haemolysed as rapidly as red cells from other strains of mice. Because of their susceptibility to tissue haemolysin, mouse red cells have been used in the haemolysis test system in most experiments since they permit haemolytic titres to be determined within a convenient period of time.

*Haemolysis of mouse red cells by rat tumour extracts*

Typical time-dilution curves for complete haemolysis of mouse red cells by rat tumour extracts are shown in Fig. 1 (*a*, *c*). The form of these curves reveals the presence of an inhibitory zone at high concentrations of extract so that on dilution,

FIG. 1.—Time-dilution curves for haemolysis of mouse red cells by transplanted rat tumour extracts.

*a* and *c*.—Typical curves showing varying amounts of lysis inhibition at high concentrations of extract.

*b*.—Curve showing abnormal production of lysin during incubation of extracts.

the activity initially increases (haemolysis occurs more rapidly), passes through a maximum and then decreases with further dilution. Inhibition zones at high concentration were observed in all the tumour extracts tested although the amount of inhibition varied considerably. In many cases, considerable inhibition was obtained so that a definite maximum activity could be determined at some dilution of extract, usually in the range 1/4 to 1/16 (Fig. 1*a*). In other experiments, slight inhibition was obtained in only one or two dilutions of extracts so that the inhibition zone appeared as a small inflexion on the time-dilution curve (Fig. 1*c*).

The inhibitory factor revealed at high concentrations of extract makes direct comparison of the activities of extracts extremely difficult. In previous studies (Tyler, 1951), the time required for complete haemolysis of red cells by a single arbitrarily fixed dilution of extract was used as a measure of haemolytic activity. Obviously this measurement does not necessarily measure the true activity of an extract since observations may be made in the inhibition zone. Determinations of the percentage haemolysis produced by various dilutions of extract after an arbitrarily fixed period of time are also unsatisfactory since the measurements may fail to reveal the inhibitory zone, whilst observations after prolonged incubation may be uncertain due to the production of fresh haemolysin from the incubated extracts (Ponder, 1951 ; Ponder and Nesmith, 1952).

The activity of extracts can be determined, however, by observing the maximum dilution of extract producing complete haemolysis at regular intervals over a period of time or preferably by determining the time required to produce complete haemolysis by dilutions of extract and constructing a time-dilution curve. If the measurements are made for a sufficient period of time, usually 3 to 5 hours, a maximum dilution titre can be determined whilst observations of the rate of haemolysis will indicate whether or not fresh haemolysin is being produced by pre-incubation effects. Thus the activity of an extract expressed as the maximum dilution producing complete haemolysis can be determined from the upper asymptote of the time-dilution curve whilst the form of the curve indicates whether or not lysin inhibitor is present and also whether pre-incubation effects are occurring. In several experiments, an increase in the slope of the time-dilution curve was observed after a certain period of time indicating an increase in the rate of haemolysis due to abnormal production of lysin (Fig. 1*b*). These results have not been recorded since interpretation of the curves is difficult.

The maximum dilution titres for haemolysis of mouse red cells by rat and mouse tumour extracts are shown in Table II. All of the transplanted rat tumour extracts were active against mouse red cells and the majority (72 per cent) had haemolytic titres of 1/64 or 1/128. Extracts of the two transplanted mouse tumours included

TABLE II.—*Haemolysis of Mouse Red Cells by Extracts of Various Transplanted Tumours*

| Tumour | Number of extracts tested. | Range | Haemolytic titres.* Number of extracts showing titres of: 4 | 8 | 16 | 32 | 64 | 128 |
|---|---|---|---|---|---|---|---|---|
| Rat— | | | | | | | | |
| Walker carcinoma | 6 | 4–128 | 1 | — | 1 | — | 2 | 2 |
| Sarcoma : | | | | | | | | |
| S5 | 6 | 4–128 | 1 | — | 1 | — | 2 | 2 |
| S66 | 15 | 32–128 | — | — | — | 4 | 7 | 4 |
| S69 | 10 | 8–128 | — | 1 | 3 | 1 | 3 | 2 |
| Mouse— | | | | | | | | |
| Strain C : | | | | | | | | |
| Sarcoma | 9 | 4–64 | 2 | 1 | 3 | 1 | 2 | — |
| Strain A : | | | | | | | | |
| Mammary carcinoma | 9 | 4–128 | 1 | 1 | 3 | 2 | 1 | 1 |

* Titres expressed as reciprocals of maximum dilution producing haemolysis.

for comparison had similar haemolytic titres although they were generally slightly less active.

*Haemolysis by extracts of normal rat tissues*

Normal rat organ extracts were feebly active against rat red cells so that haemolysis only occurred in dilutions up to 1/4 even after incubation at 37° C. for 24 hours or longer. The extracts showed considerably more activity against mouse red cells producing haemolysis at dilutions up to 1/64 following incubation for 3 to 5 hours (Table III).

TABLE III.—*Haemolytic Activity of Normal Rat Tissue Extracts Against Rat and Mouse Red Cells*

| Organ | Number of extracts tested* | Number showing haemolysis at dilutions of: 1/2 | 1/4 | 1/8 | 1/16 | 1/32 | 1/64 |
|---|---|---|---|---|---|---|---|
| | (a) Rat red cells : 24-hour titre | | | | | | |
| Liver | 5/5 | 3 | 2 | — | — | — | — |
| Lung | 5/5 | 1 | 2 | 1 | 1 | — | — |
| Kidney | 1/5 | 1 | — | — | — | — | — |
| Spleen | 4/5 | 1 | 3 | — | — | — | — |
| Heart | 0/5 | | | | | | |
| Serum | 0/5 | | | | | | |
| | (b) Mouse red cells : 5-hour titre | | | | | | |
| Liver | 13/15 | — | 2 | 7 | 4 | — | — |
| Lung | 15/15 | — | 2 | 4 | 6 | 2 | 1 |
| Kidney | 14/15 | — | 3 | 3 | 8 | — | — |
| Spleen | 15/15 | — | — | 1 | 3 | 8 | 3 |
| Heart | 0/15 | | | | | | |
| Serum | 0/15 | | | | | | |

* Numerator—Number of extracts causing haemolysis. Denominator—Number of extracts tested.

All organ extracts, except those of heart tissue, possessed haemolytic activity, However, serum inactivated at 56° C. was completely inactive even after prolonged incubation. Heating at 56° C. did not destroy the activity of other tissue extracts. Spleen extracts possessed the greatest activity although in two experiments, lung extracts showed comparable activity. However, extracts of other organs were generally 2 to 4 times less active than spleen extracts. The time-dilution curves for complete haemolysis of mouse red cells by rat organ extracts (Fig. 2) reveal that in addition to being the most active, spleen extracts produce haemolysis more rapidly than any other organ extract. Thus, the maximum dilution of spleen extract showing activity produces haemolysis in a much shorter time than any of the other organ extracts at their maximum active dilution.

Inhibitory zones were again observed at high concentrations of organ extract although the amount of inhibition generally was much lower than that detected in tumour extracts. The amount of inhibition produced by various organ extracts also varied considerably, liver extracts showing the most and spleen extracts the least.

### DISCUSSION

The results indicate that haemolysins can be isolated in saline extracts of normal rat tissues as well as from transplanted rat tumour tissue. These haemolysins are not species-specific since under the right conditions, they will haemolyse the red cells of several species. However, the mouse red cell appears to be very much more susceptible to this type of haemolysin than any of the other red cells tested. This may explain why in the earlier studies (Gross, 1948), it was claimed that mouse tumour haemolysin was species-specific, since with an insensitive technique for measuring haemolysis, red cells other than those of the mouse would appear to be resistant to the haemolysin. In fact the findings of Gross (1948) merely reflect the relative susceptibility *in vitro* of the mouse red cell to this particular type of haemolytic system. That varying red cell resistance series exist for different haemolysins is illustrated by the findings of Howard and Wallace (1953) who showed that the mouse red cell showed exceptional resistance to haemolysis by streptolysin O.

Fig. 2.—Typical time-dilution curves for haemolysis of mouse red cells by normal rat organ extracts.

Spleen ○—○

Lung ●—●

Kidney ×—×

Liver △—△

The relative resistance of rat red cells to haemolysis may be due to the fact that they are readily agglutinated by the tissue extracts. This would have the effect of making the red cells more resistant to the haemolysin. Haemagglutination activity has previously been observed by Salaman (1948) in extracts of a variety of tumours against mouse and rabbit red cells and by Gross (1948) and Ponder and Nesmith (1952) for rat and mouse tumour extracts against their homologous red cells. As suggested by Ponder and Nesmith (1952), agglutination and haemolysis may be reciprocally related, the former occurring when the other does not and vice versa. It is perhaps significant that extracts of the Walker carcinoma which were much more haemolytic for rat red cells than any of the other rat tumour extracts tested, caused no haemagglutination.

In confirmation of the findings of Ponder and Nesmith (1952), atypical time-dilution curves were obtained for complete haemolysis of mouse red cells by both normal and malignant rat tissue extracts (Fig. 1 and 2), indicating the presence of an inhibitory factor at high concentrations of extract. This inhibitory factor makes direct comparison of the activities of extracts extremely difficult, The haemolytic activity of an extract can be expressed, however, as the maximum dilution producing haemolysis of a standard amount of red cells under fixed conditions of time and temperature, providing that no abnormal lysin production is occurring. This dilution titre will measure the maximum amount of haemolysin becoming available from the extract under the conditions used and will depend upon a number of competing factors such as the rates of inactivation of lysin and inhibitor and the rate of lysin production or activation from inactive or slightly active lysin complexes.

The haemolytic activities of various normal rat organ extracts were found to vary considerably ; spleen having the highest activity whilst serum and heart extracts were inactive. Studies of the rates of haemolysis by organ extracts indicated that in addition to being the most active, spleen extracts also produced haemolysis most rapidly indicating that the lysin was more readily available than that in other normal organ extracts. This may well be due to the absence of inhibitory substances since spleen extracts rarely showed inhibitory zones in time-dilution curves.

Comparison of the results obtained with extracts of normal and malignant rat tissue extracts (Tables II and III) indicates that the haemolytic activity of transplanted tumour extracts was generally 2 to 4 times greater than that of the most active normal tissue extract, i.e. spleen. Since the haemolytic system in rat tissue extracts consists of a mixture of lysins and inhibitors, the increased activity of tumour extracts may be due to an actual increase in lysin or to a decrease in inhibitor concentration. Although no quantitative data have been obtained regarding the variations of inhibitor, comparison of the time-dilution curves for haemolysis by extracts of normal organs; particularly spleen, and transplanted tumours indicated that the more active tumour extracts also showed the most inhibition. Thus, it would seem that there is an actual increase in the haemolysin content of tumour extracts.

Attempts to obtain evidence relating tumour tissue haemolysins to a humoral haemolytic factor so far have been unsuccessful since sera from tumour-bearing animals have not shown any *in vitro* haemolytic activity. This is not surprising however, since in the majority of previous studies on normal serum haemolysins, active extracts could only be prepared in non-aqueous solvents and it was suggested that lysin-inhibitors may be responsible for the *in vitro* inactivity of whole serum (Ponder, 1952*b*). These inhibitory substances are highly active *in vitro* and can inhibit the activity of a variety of haemolysins, many of which are far more active than the naturally occurring tissue haemolysins (Ponder, 1952b ; Stollerman, 1954). Therefore, it will be necessary to separate the naturally occurring serum lysins from the inhibitors before any conclusive evidence can be obtained regarding the relationship between serum and tissue haemolysins.

SUMMARY

1. Extracts of normal rat organs and transplanted tumours have been shown to contain a water soluble haemolytic factor. These haemolysins were not species-specific since they produced haemolysis of the red cells of several species including rat, mouse, rabbit, guinea-pig and sheep. However, the mouse red cell was very much more susceptible to the haemolysins than any of the other red cells tested. Extracts were only weakly haemolytic against homologous rat red cells although they readily caused haemagglutination.

2. Abnormal time-dilution curves for haemolysis of mouse red cells were obtained with extracts of both normal and malignant rat tissue extracts, indicating the presence of an inhibitory zone at high concentrations of extract. It is concluded that the haemolytic system consists of a mixture of lysins and inhibitors.

3. The haemolytic activity of normal tissue extracts varied considerably; spleen showing the greatest activity whilst heart extracts and serum were inactive. The haemolytic system in spleen extracts appears to be different from that of other normal tissue extracts. This is probably because spleen extracts contain little or no lysin inhibitor.

4. The haemolytic activity of transplanted tumour extracts was usually 2 to 4 times greater than that of the most active normal tissue extract, i.e. spleen. This increase in activity is probably due to an increase in haemolysin content rather than to a decrease in inhibitor.

Thanks are due to Dr. A. L. Walpole, Imperial Chemical (Pharmaceuticals) Ltd. for supplying the tumour-bearing mice and to Miss M. E. Gillard for skilled technical assistance. This work was supported by the Nottinghamshire Council of the British Empire Cancer Campaign.

REFERENCES

ADELSBERGER, L.—(1951) *Cancer Res.*, **11**, 658.
BALDWIN, R. W.—(1955) *Brit. J. Cancer*, **9**, 646.
GROSS, L.—(1948) *J. Immunol.*, **59**, 173.—(1949) *Proc. Soc. exp. Biol., N.Y.*, **70**, 656.
HOWARD, J. G. AND WALLACE, K. R.—(1953) *Brit. J. exp. Path.*, **34**, 181.
HYMAN, G. A. AND HARVEY, J. L.—(1954) *Blood*, **9**, 911.
*Idem*, GELLHORN, A. AND HARVEY, J. L.—(1956) *Ibid.*, **11**, 618.
LASER, H.—(1950) *J. Physiol.* **110**, 338.
MAEGRAITH, B. G., MARTIN, N. H. AND FINDLAY, G. M.—(1943) *Brit. J. exp. Path.*, **24**, 58.
MILLER, A., CHODOS, R. B., EMERSON, C. P. AND ROSS, J. F.—(1956) *J. clin. Invest.*, **35**, 1248.
PONDER, E.—(1951) *J. gen. Physiol.*, **34**, 551.—(1952*a*) *Rev. Hémat.*, **7**, 436.—(1952*b*) *J. gen. Physiol.*, **35**, 361.
*Idem* AND NESMITH, J.—(1952) *Cancer Res.*, **12**, 104.
SALAMAN, M. H.—(1948) *Brit. J. Cancer*, **2**, 253.
SHEETS, R. F., HAMILTON, H. E., DEGOWIN, E. L. AND JANNEY, C. D.—(1954) *J. clin. Invest.*, **33**, 179.
SINGER, K.—(1940) *Amer. J. med. Sci.*, **199**, 466.
STOLLERMAN, G. H.—(1954) *J. clin. Invest.*, **33**, 1233.
TYLER, D. B.—(1951) *Amer. J. Physiol.*, **164**, 467.
WEIL, R.—(1907) *J. med. Res.*, **16**, 287.